# Peripheral nerve biopsy in pure neural leprosy: a 26-year experience in Brazil

**DOI:** 10.1093/braincomms/fcag249

**Published:** 2026-06-26

**Authors:** Ana C Siquara-de-Sousa, Mariana Hacker, Robson T Vital, Izabela J R Pitta, Clarissa Spitz, Cristiane C Domingues, Anna M Sales, Leila Chimelli, Sergio L G Antunes, Euzenir N Sarno, Roberta O Pinheiro, Marcia R Jardim

**Affiliations:** Leprosy Laboratory, Oswaldo Cruz Institute, Oswaldo Cruz Foundation (FIOCRUZ), Rio de Janeiro 21040-360, Brazil; Post-Graduate Program in Neurology, Federal University of the State of Rio de Janeiro, Rio de Janeiro 20270-004, Brazil; Leprosy Laboratory, Oswaldo Cruz Institute, Oswaldo Cruz Foundation (FIOCRUZ), Rio de Janeiro 21040-360, Brazil; Leprosy Laboratory, Oswaldo Cruz Institute, Oswaldo Cruz Foundation (FIOCRUZ), Rio de Janeiro 21040-360, Brazil; Department of Neurology, Pedro Ernesto University Hospital, Rio de Janeiro State University, Rio de Janeiro 20551-900, Brazil; Leprosy Laboratory, Oswaldo Cruz Institute, Oswaldo Cruz Foundation (FIOCRUZ), Rio de Janeiro 21040-360, Brazil; Department of Internal Medicine, Neurology, Fluminense Federal University, Niterói 24033-900, Brazil; Leprosy Laboratory, Oswaldo Cruz Institute, Oswaldo Cruz Foundation (FIOCRUZ), Rio de Janeiro 21040-360, Brazil; Leprosy Laboratory, Oswaldo Cruz Institute, Oswaldo Cruz Foundation (FIOCRUZ), Rio de Janeiro 21040-360, Brazil; Leprosy Laboratory, Oswaldo Cruz Institute, Oswaldo Cruz Foundation (FIOCRUZ), Rio de Janeiro 21040-360, Brazil; Paulo Niemeyer State Brain Institute, Rio de Janeiro 20230-024, Brazil; Leprosy Laboratory, Oswaldo Cruz Institute, Oswaldo Cruz Foundation (FIOCRUZ), Rio de Janeiro 21040-360, Brazil; Leprosy Laboratory, Oswaldo Cruz Institute, Oswaldo Cruz Foundation (FIOCRUZ), Rio de Janeiro 21040-360, Brazil; Leprosy Laboratory, Oswaldo Cruz Institute, Oswaldo Cruz Foundation (FIOCRUZ), Rio de Janeiro 21040-360, Brazil; Rio de Janeiro Research Network on Neuroinflammation, Oswaldo Cruz Institute, Oswaldo Cruz Foundation, Rio de Janeiro 21040-360, Brazil; National Institute of Science and Technology on Neuroimmunomodulation, Oswaldo Cruz Institute, Oswaldo Cruz Foundation (FIOCRUZ), Rio de Janeiro 21040-360, Brazil; Leprosy Laboratory, Oswaldo Cruz Institute, Oswaldo Cruz Foundation (FIOCRUZ), Rio de Janeiro 21040-360, Brazil; Post-Graduate Program in Neurology, Federal University of the State of Rio de Janeiro, Rio de Janeiro 20270-004, Brazil; Department of Neurology, Pedro Ernesto University Hospital, Rio de Janeiro State University, Rio de Janeiro 20551-900, Brazil; Rio de Janeiro Research Network on Neuroinflammation, Oswaldo Cruz Institute, Oswaldo Cruz Foundation, Rio de Janeiro 21040-360, Brazil; National Institute of Science and Technology on Neuroimmunomodulation, Oswaldo Cruz Institute, Oswaldo Cruz Foundation (FIOCRUZ), Rio de Janeiro 21040-360, Brazil

**Keywords:** peripheral nervous system diseases, sural nerve, differential diagnosis, database

## Abstract

Brazil ranks second globally in leprosy burden, with approximately 25 000 cases reported in 2022. This study aimed to analyse the 26-year experience (1997–2023) in nerve biopsies at a specialized leprosy outpatient clinic, focusing on their usefulness in diagnosing peripheral neuropathy in patients with clinical and neurophysiological features suggestive of leprosy but lacking dermatological lesions. Tissue samples were preserved in glutaraldehyde, buffered formalin and liquid nitrogen, with histopathological analysis using various staining techniques and molecular testing on frozen material. In 819 cases, 529 were diagnosed with leprosy (64.6% overall leprosy diagnosis rate), and 207 pure neural leprosy cases were confirmed. Other conditions identified included vasculitis, diabetic neuropathy and amyloidosis, while 16.5% of cases yielded inconclusive results. The predominant clinical symptoms included sensory disorders (85.7%), localized paraesthesia (70.3%) and muscle weakness (59.4%), with multiple mononeuropathies frequently observed on electroneuromyography. The most biopsied nerves were the ulnar cutaneous branch (50.6%), sural (37.2%) and superficial peroneal (6.8%). Nerve biopsy proved valuable for confirming leprosy and distinguishing differential diagnoses in complex cases, particularly when clinical findings alone were insufficient. Integrating clinical–pathological correlation optimized diagnostic accuracy, underscoring the importance of this tool in managing suspected neural leprosy and guiding treatment in challenging cases.

## Teaser

Siquara-de-Sousa *et al*. reported that nerve biopsy provides critical diagnostic confirmation in patients with peripheral neuropathy suspected of pure neuritic leprosy. By analysing a large 26-year cohort, they show that biopsy remains essential when clinical, electrophysiological or imaging findings are inconclusive, reinforcing its role in complex diagnostic pathways.

## Graphical Abstract

**Graphical Abstract:**
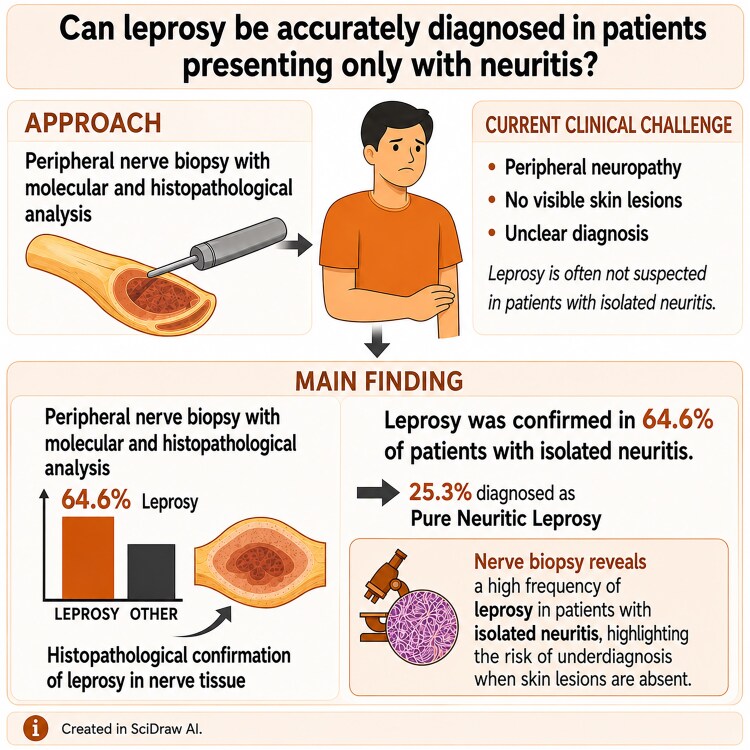

## Introduction

Leprosy is a chronic infectious disease caused by *Mycobacterium leprae* or *M. lepromatosis*, characterized by its multi-systemic involvement, particularly affecting the skin and peripheral nerves.^1,2^ Despite significant advances in treatment and disease control, leprosy continues to pose a substantial public health challenge in endemic regions.^3^ While skin and nerve manifestations typically coexist, a subset of patients presents with neurological symptoms in the absence of visible dermatological alterations, a condition termed pure neuritic leprosy (PNL).^4^

Diagnosing leprosy-related neuropathy, especially PNL, remains challenging. Histopathological examination of a peripheral nerve biopsy is considered the gold standard for diagnosing PNL, as it allows direct observation of pathological changes and the detection of *M. leprae*. However, this method is invasive, technically demanding and limited by accessibility to certain sensory nerves. Furthermore, *M. leprae* may not always be detected, and histological findings can be non-specific, making definitive diagnosis difficult. Despite these limitations and the urgent need for improved diagnostic tools and strategies, nerve biopsies appear to contribute to the early diagnosis of leprosy neuropathy.^4-8^

Patients with PNL often face delays in diagnosis due to their initial presentation. Unlike cases with skin lesions, which are promptly referred to dermatology, patients with primarily neurological symptoms frequently seek care in neurology, when leprosy may not be immediately considered. Such delays underscore the importance of increasing awareness and refining diagnostic approaches for PNL.^1,9^

Advances in molecular techniques, such as PCR, and research into genetic and biomolecular signatures are beginning to transform the diagnostic landscape of leprosy neuropathy.^10^ These tools hold promise for enhancing sensitivity and specificity, potentially reducing reliance on invasive procedures like nerve biopsies. However, in cases of PNL where clinical and laboratory evaluations are inconclusive, nerve biopsy remains a critical diagnostic tool, providing clarity in distinguishing leprosy neuropathy from other conditions such as vasculitis, amyloidosis or paraneoplastic syndromes.^11,12^

Here, we evaluated the diagnostic value of nerve biopsy in patients with suspected peripheral neuropathies, focusing on leprosy. The study also aimed to investigate the relationship between patient demographics, clinical presentation and diagnostic outcomes, including histopathological and electrophysiological results, by reviewing nerve biopsy records in a leprosy referral centre in Brazil.

## Materials and methods

A retrospective analysis of nerve biopsies from the last 26 years (1997–2023) was conducted at the Souza Araujo Outpatient Unit (Leprosy Laboratory, Oswaldo Cruz Institute, Fiocruz), a referral centre of the Brazilian Ministry of Health for leprosy treatment and management.

From 1997 to December 2023, patients suspected of having PNL were evaluated at the Leprosy Outpatient Clinic of the Oswaldo Cruz Foundation in Rio de Janeiro, Brazil. Clinical and electrophysiological examinations were performed before initiating the study.

The neurological clinical examination assessed initial neurological symptoms such as weakness, sensory changes and autonomic dysfunction. It also evaluated the presence of erythrocytosis, paraesthesia, peripheral nerve thickening, pain, reflexes, vibratory sensory changes and other sensory and muscle strength alterations (using the British Medical Research Council scale).

Sensory nerve conduction studies (NCS) were routinely conducted on the median, radial, ulnar, sural and peroneal superficial nerves, and motor NCS was conducted on the median, ulnar, deep peroneal and tibial nerves. Clinical and neurophysiological studies showed abnormalities in all patients who underwent a biopsy, with abnormal sensory conduction being a criterion for the examination.

Patients were excluded from the study if they had skin patches or lesions, or a history of skin conditions. Other exclusion criteria included diabetes mellitus, alcoholism, hepatitis B or C, HIV or HTLV-I infections and rheumatic diseases. The study was conducted following international ethical standards and was approved by the Ethics Committee of the Oswaldo Cruz Foundation. All participants provided written consent.

Over the past 26 years, an average of 31 nerve biopsies was conducted annually, with no observable decline in procedure frequency during this period. The mean number of biopsies has remained stable in recent years, totalling 35 in 2021, 40 in 2022 and 43 in 2023. Despite limitations imposed by the COVID-19 pandemic in 2020, when only 11 biopsies were performed, subsequent annual figures indicate a consistent trend in the volume of procedures.

For nerve biopsy processing, each biopsy fragment was divided into two parts and fixed in 2.5% glutaraldehyde for Araldite embedding, 10% Millonig’s neutral buffered formalin for paraffin embedding and frozen in liquid nitrogen. Paraffin blocks were sectioned at a microtome (Thermo Fisher Scientific—Thermo Shandon, Massachusetts, USA) into 5-μm-thick sections. The sections were laid on glass slides, had their paraffin removed via serial xylene and alcohol immersion, were rehydrated and then stained with haematoxylin-eosin to evaluate the inflammatory infiltrate and cell populations, by using Gomori’s trichrome to assess fibrosis and nerve structure and Wade staining to detect acid-fast bacilli. Part 2 of the nerve samples was fixed in 2.5% glutaraldehyde, washed in sodium cacodylate buffer, post-fixed in 2% osmium tetroxide, washed again in the same buffer, dehydrated in serial-graded acetone batches, impregnated and included in epon. The blocks were sectioned into 0.5-μm-thick sections (ultramicrotome from Reichert, New York, USA) and sections were stained with Toluidine Blue. Histopathological and special staining techniques have not changed over the 26-year period. The material in liquid nitrogen was preserved for molecular testing and research projects, as previously described.^13,14^

In addition to the database information, predetermined histopathological changes were recorded, and a final histopathological report was issued for each examined nerve. A total of 31 histopathological parameters were studied (Supplementary Table 1), which were defined by the team of pathologists based on the literature (mainly on leprosy reports), such as the work of Weis *et al*. and others.^13-16^ The diagnostic thresholds have been updated in line with established literature, but without significant changes. Furthermore, the reporting formats have not undergone major alterations over time.

Biopsy protocols were consistent, conducted by the same physician and based on established practices. Diagnostic and pathological assessments followed standardized, literature-based criteria, with all team members trained by a senior pathologist. Histopathological evaluations were also performed using standardized procedures. The pathologists had prior access to primary findings from neurological assessments, electrophysiological examinations and diagnostic hypotheses, like a real-world diagnostic workflow in a referral centre.

All data were systematically documented in designated medical records and subsequently incorporated into the institution’s database. Unavailable information was classified as ‘not recorded’ in Table 1. For the clinical condition section, patients without recorded data were excluded from the analysis.

**Table 1:** Patients’ parameters (850 nerve biopsies, except*)

Evaluated parameters	% (*n*)	95% CI^a^
Gender		
Male	61.8% (525)	58.4–65.1
Female	36.4% (309)	33.1–39.7
Not registered	1.9% (16)	1.08–3.03
Age		
11–19 years	2.8% (24)	1.8–4.2
20–29 years	6.2% (53)	4.7–8.1
30–39 years	14.5% (123)	12.1–17.0
40–49 years	17.5% (149)	15.2–20.2
50–59 years	21.1% (179)	18.4–23.9
60–69 years	16.2% (138)	13.8–18.9
70–79 years	9.2% (78)	7.3–11.3
80–89 years	2.5% (21)	1.5–3.7
Not registered	10% (85)	8.1–12.2
Nerve biopsy		
Ulnar	50.6% (430)	47.2–53.9
Sural	37.2% (316)	33.9–40.5
Peroneal (fibular)	6.8% (58)	5.2–8.7
Median	0.8% (7)	0.3–1.7
Radial	0.7% (6)	0.2–1.5
Other	0.4% (3)	0.07–1.03
Not registered	3.5% (30)	2.4–5.0
Clinical condition (683 patients)		
Sensory disorder (localized)	85.7% (585)	
Paraesthesia (localized)	70.3% (480)	
Low muscular strength	59.4% (406)	
Nerve thickening	41.4% (283)	
Motor disorder	37.2% (254)	
Erithrocyanosis	34.6% (236)	
Pain (localized)	30.5% (208)	
Paraesthesia	30.5% (208)	
Sensory disorder	14.9% (102)	
Paresia	13.6% (93)	
Pain	10.1% (69)	
Dysautonomia	0.1% (1)	
Diagnosis* (819 biopsies)		
Leprosy	64.6% (529)	61.2–67.9
Pure neural leprosy	25.3% (207)	22.3–28.4
Not registered	15.3% (125)	12.9–17.9
Granuloma or caseous necrosis	11.5% (94)	9.4–13.8
Neuritis	6.7 (55)	5.1–8.6
Disease relapse	4.6% (38)	3.3–6.3
Reactional state (neuritis)	1.2% (10)	0.6–2.2
Descriptive report	16.5% (135)	14.0–19.2
Vasculitis	4.5% (37)	3.2–6.2
None alteration	3.8% (31)	2.6–5.3
Diabetic neuropathy	3.7% (30)	2.5–5.2
Compression neuropathy	2.4% (20)	1.5–3.7
Amyloidosis	2% (17)	1.2–3.3
CIDP	0.7% (6)	0.3–1.6
Other^b^	1.7% (14)	0.9–2.8

*n* = sample size. ^a^*P* < 0.05. ^b^Alcoholic neuropathy, multifocal acquired demyelinating sensory and motor neuropathy, among rarer diagnoses.

### Statistical analysis

Data were analysed using descriptive statistics. Continuous variables, such as patient age, were summarized using mean, median and range. Categorical variables—including demographic data, clinical features, electrophysiological findings and histopathological diagnoses—were presented as absolute frequencies and percentages, with corresponding 95% confidence intervals (CIs). A significance level of *P* < 0.05 was applied to the calculation of confidence intervals. The analysis included all available data from the institutional database. Due to the retrospective design of this study, the analyses were predominantly descriptive, and formal inferential statistical comparisons between groups were not performed. Several STROBE checklist items were designated as ‘Not Applicable’ due to the absence of multivariable modelling or hypothesis-testing analyses within this descriptive study design.

## Results

A total of 850 nerve biopsy records were reviewed. In 31 biopsies (3.6%), no peripheral nerve tissue was identified (Supplementary Fig. 1). These cases corresponded to patients with marked fibrosis in the affected limb, which made nerve localization and sampling technically challenging.

The male-to-female ratio was 1.7:1, with 525 biopsies performed in men and 309 in women. Patient ages ranged from 11 to 89 years, with both the mean and median age being 51 years. The most frequently biopsied age groups were 50–59 years (21.1%), followed by 40–49 years (17.5%) and 60–69 years (16.2%) (Table 1).

Among the 819 biopsies in which nerve tissue was present, 529 cases (64.6%) were diagnosed with leprosy. The disease was confirmed in 207 patients previously suspected of having PNL, and 150 cases were positive for bacilli on acid-fast Wade staining. Microfasciculation of the endoneurium was observed in 63 cases on semi-thin sections. Other diagnoses included vasculitis (4.5%), diabetic neuropathy (3.7%), compression neuropathy (2.4%), amyloidosis (2%), chronic inflammatory demyelinating polyneuropathy (CIDP) (0.7%), alcoholic neuropathy (0.4%), multifocal acquired demyelinating sensory and motor neuropathy (0.2%), among other rare conditions. No peripheral nerve tumours were identified (Table 1 and Supplementary Fig. 1).

On average, 31 nerve biopsies were performed annually, with no significant reduction in the number of procedures over the years. The annual average remained stable during the last five years, even during the COVID-19 pandemic, despite restrictions on outpatient evaluations.

The high number of leprosy diagnoses reflects the referral nature of our centre, where biopsies are frequently performed in patients with long-standing disease (more than five years of treatment), worsening neurological function or recurrent reactions. Nerve biopsy was also decisive in confirming PNL in patients without dermatological lesions, where diagnosis had not been achieved through other methods such as physical examination, slit-skin smear, PCR of skin samples or ultrasound. Additional indications for biopsy included clinical suspicion of vasculitis or amyloidosis (both prevalent in the country), PNL cases with abnormal NCS, lack of response to initial therapy and situations in which imaging, electrophysiology, serology or molecular tests yielded inconclusive results.

The nerves selected for biopsy were those most accessible and those demonstrating abnormalities on physical examination, imaging or EMG. Because patients with suspected leprosy often present symptoms in the extremities, the cutaneous branch of the ulnar nerve was the most frequently biopsied (50.6%), which also contributed to the higher number of leprosy diagnoses in this location. The sural nerve was biopsied in 37.2% of cases, and the superficial peroneal nerve in 6.8% (Table 1). When comparing the proportion of confirmed leprosy cases among biopsies of the cutaneous branch of the ulnar nerve and the sural nerve, no significant difference was observed.

The most common presenting symptoms were paraesthesia (30.5%), localized pain (30.5%), sensory disturbances (14.9%) and paresis (13.6%). Neurological examination findings were highly consistent with patient complaints, including localized sensory deficits (85.7%), localized paraesthesia (70.3%) and reduced muscle strength (59.4%). Nerve thickening was less frequent than expected, occurring in 41.4% of cases, despite being a classical finding in leprosy (Table 1).

EMG abnormalities were present in all patients who underwent biopsy and consistently demonstrated sensory conduction impairment. The most frequent EMG patterns were multiple mononeuropathy (94.1% of sensorimotor nerves, 82.8% of motor nerves and 71.7% of sensory nerves) and mononeuropathy (8.4% of motor nerves and 9.9% of sensory nerves). Sensory–motor polyneuropathy was identified in only 3.4% of cases.

**Figure 1:**
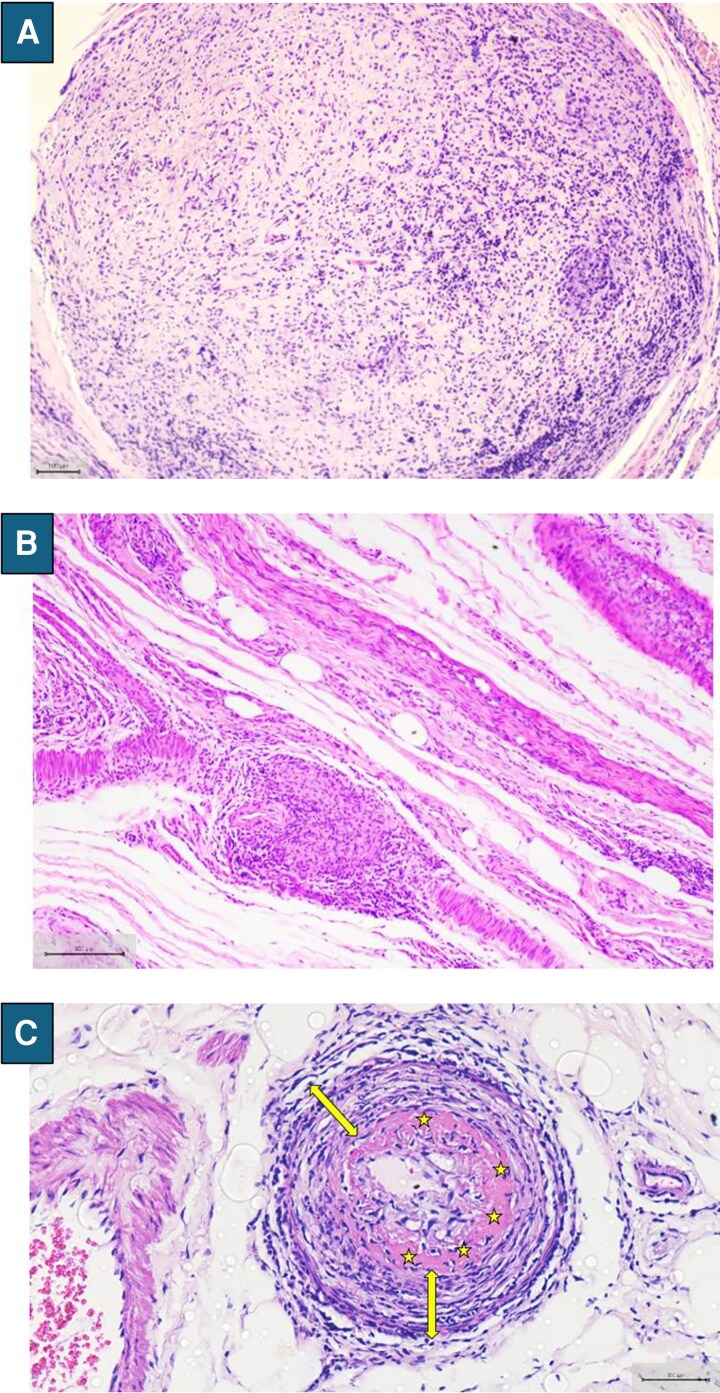
**Histopathological features of leprosy in peripheral nerves (A) Leprosy.** Haematoxylin and eosin, 100×. In the perineurium and endoneurium, there are several foci of marked mononuclear inflammatory infiltrate (with macrophages). (**B**) Leprosy. Haematoxylin and eosin, 200×. In the epineurium, there are several foci of mild to moderate mononuclear inflammatory infiltrate (with macrophages), thickening of the wall of some vessels and outlines of granuloma (absence of caseous necrosis). (**C)** Vasculitis. Haematoxylin and eosin, 200×. Inflammatory cells are observed in the vessel wall (arrow) and leukocytoclasia (fragmentation of inflammatory cell nuclei, extravasation of red blood cells and fibrinoid necrosis (*).

Histopathology was non-specific in 135 cases (16.5%), resulting in descriptive reports without a definitive diagnosis. In 31 biopsies, no histopathological abnormalities were found. Representative histological findings are shown in Fig. 1A–C, including inflammatory infiltrates typical of leprosy (A–B) and vasculitis (C).

## Discussion

Peripheral nerve biopsy has been used for decades as a diagnostic technique to evaluate neuropathies when other diagnostic options were inconclusive, as well as its yield in the evaluation of peripheral neuropathies.^17-23^ Nerve biopsy is generally indicated for inflammatory diseases or diseases with potential for treatment, such as vasculitis, neurolymphomatosis, primary neoplasm of the peripheral nerve and in countries in which leprosy is frequent, to confirm the diagnosis of PNL. In these cases, it has been pivotal for diagnosis.^12,24,25^ It is also relevant for diseases like amyloidosis and neurosarcoidosis, as well as for differential diagnosis and subtypes of CIDP.^12,24-26^

Nerve biopsy should be prioritized in the diagnostic approach to peripheral neuropathies when histopathological confirmation is expected to alter clinical management or when non-invasive modalities yield inconclusive results. Unlike molecular or imaging techniques, nerve biopsy provides direct visualization of axonal injury, demyelination, inflammatory infiltrates, vasculitic changes or amyloid deposition. These features are often essential for distinguishing among inflammatory, infiltrative or infectious aetiologies. It remains particularly valuable in suspected cases of vasculitic, amyloid or infectious neuropathies, where tissue examination can confirm the diagnosis and guide targeted therapy.^12,24,25^ While advances in serological, molecular and neuroimaging methods have expanded diagnostic options, nerve biopsy retains an indispensable role as a complementary and confirmatory tool in complex or atypical neuropathies.

However, histopathological studies are not always able to define the aetiology of neuropathy. In a retrospective analysis by Prada *et al*.^27^ (1981–2017), which included 1179 sural nerve biopsies, 53% of cases had a definitive diagnosis. As in other retrospective analyses with an equivalent or smaller number of cases, the most frequently diagnosed disease was vasculitis.^23,27-36^ Other diseases frequently found were amyloidosis, acquired demyelinating neuropathy (CIDP and anti-MAG) and hereditary neuropathy (Charcot-Marie-Tooth disease and subtypes). Leprosy represented a small number of cases even in an analysis carried out in India, the country with the highest prevalence worldwide.^37^ In previous studies, the diagnosis of leprosy neuritis was made in 3.97% of the total nerve biopsy cases (453 biopsies), using the same diagnostic criteria as Antunes and colleagues.^16,38,39^

Although molecular assays such as PCR and neuroimaging techniques (e.g. MRI neurography, high-resolution ultrasound) have expanded the diagnostic armamentarium for peripheral neuropathies, nerve biopsy remains a critical tool in specific clinical contexts, as it helps in defining the aetiology.^40^ In this study, histological patterns (Fig. 1) corresponded with established features of leprosy neuropathy, supporting the diagnostic utility of biopsy. Its use should be reserved for cases in which less invasive modalities fail to provide a definitive diagnosis or when histopathological information is essential to guide management.

The high percentage of leprosy diagnoses in our cohort could be due to the fact that our facility serves as a referral centre and receives many cases of suspected PNL. Patients with non-specific neuropathy complaints are referred for nerve biopsy when there is no good correlation between the results of complementary exams and the clinical condition. There were 135 cases (16.5%) with descriptive reports but without a diagnostic conclusion, a percentage similar to that obtained in the literature.^24,26,27,29,33^ In such situations, patients are referred to the original service, as the objective of the nerve biopsy was to rule out the diagnosis of leprosy. In 31 (3.6%) nerve biopsies, no histopathological alterations were found.

In this retrospective analysis, 64.6% of the cases were diagnosed as leprosy, reflecting the extensive experience in leprosy care and research at the referral centre. Therefore, the biopsy was essential to identify these cases, as it demonstrates histopathological patterns and allows for early initiation of treatment. In our practice, biopsy helps avoid false-positive cases in which the patient undergoes long-term treatment without correct multidisciplinary follow-up. Histological patterns (Fig. 1A–C) corresponded with established features of leprosy neuropathy, supporting the diagnostic utility of biopsy.

In addition, the Outpatient Unit uses the PGL-1 test for measuring the exposure to *M. leprae* and identifying potential cases among healthy contacts. PGL-1 is an ELISA test that detects IgM antibodies against a specific antigen of *M. leprae* (anti-phenolic glycolipid 1), and there is a positive correlation between antibody levels and the bacterial index, making this test useful for diagnosing PNL when nerve biopsies are unavailable. Our observations indicate that, at the start of treatment, patients who test positive for PGL-1 tend to be more severely affected.^41^ Although PGL-1 has high specificity (>80%) and low sensitivity (<50%), it should be combined with other strategies to determine the need for prophylactic treatment in these contacts.^42,43^ In our experience, PGL-1 is valuable for screening, as it enables a five-year follow-up of positive contacts.

Over 26 years, 131 patients underwent the PGL-1 test and subsequently had nerve biopsies for diagnostic confirmation. Among these patients, 72 tested positive for PGL-1 (55%). However, as the test primarily indicates exposure rather than confirmed infection, only 17.6% (23 patients) received a definitive leprosy diagnosis based on histopathological and clinical criteria. The remaining cases had other diagnoses as usual, like vasculitis, diabetic neuropathy or a descriptive report. Notably, an equal number of patients (23) with negative PGL-1 results were diagnosed with leprosy using the same rigorous histopathological and clinical standards.^39^ Thus, it is evident that the PGL-1 test has limitations in sensitivity when identifying patients with the disease based on nerve biopsies. Although the PGL-1 antigen plays an important role in detecting the humoral immune response against *M. leprae*, its diagnostic utility as a standalone tool is constrained by reduced sensitivity, particularly in paucibacillary or early-stage presentations. However, despite its low sensitivity, the PGL-1 test is highly specific; therefore, positive results strongly support the diagnosis of leprosy and can be considered true positives. PGL-1 positivity is intended to support, but not substitute for, thorough clinicopathological evaluation, particularly in cases of PNL or early/paucibacillary disease. For these reasons, PGL-1 should be interpreted with caution and regarded as a complementary diagnostic marker, enhancing diagnostic accuracy when used alongside clinical, serological or molecular assays, rather than serving as the sole indicator of infection.

The most common clinical presentation was a patient with sensory disorders, especially paraesthesia. This situation is typical of patients with PNL, who, for the most part, do not have pain, thickening or neuritis, but rather sensory changes. In these cases, the most common finding in NCS is the mononeuropathy multiplex. However, we also receive patients with clinical suspicion of neuritis (nerve inflammation with pain, sensory impairment and local thickening) that may be caused by leprosy, although the signs and symptoms are somewhat diverse.^44^ Nerve biopsy, therefore, helped to establish a definitive diagnosis in these cases.^16^ EMG was altered in all patients (abnormal conduction), which is a criterion for recommending a biopsy.

Though biopsies were performed at a leprosy referral centre, there were a variety of other diagnoses. Vasculitis accounted for a significant number of confirmed cases, with 5.4%. As vasculitis is one of the main indications for nerve biopsy, confirmation by this test is common. Compression neuropathy, diabetic neuropathy and amyloidosis represent 8.2% of total cases and were mainly confirmed as differential diagnoses.

Nerve biopsy plays a crucial role in distinguishing leprosy and amyloidosis. While both conditions may present similar symptoms such as painful neuropathy or trophic changes, the absence of clinical signs of dysautonomia, an asymmetric neuropathy pattern and the histopathological differences can help to differentiate them.^45^ While the number of biopsies for suspected amyloidosis has decreased due to genetic studies and the availability of salivary gland biopsies, a retrospective analysis revealed 17 confirmed cases (2%).

The limitations of this study include its retrospective design, incomplete data collection and the evolving nature of diagnoses over a 26-year period (such as advancements in genetic testing and identification of node and paranode neuropathies). Selection bias may have influenced the elevated rate of leprosy diagnosis, as a referral centre, a higher proportion of leprosy cases was anticipated. Furthermore, the adoption of novel examination techniques within the outpatient clinic contributed to enhanced diagnostic accuracy.

In our experience, nerve biopsies were most beneficial in cases with well-defined clinical suspicion, aligning with the literature. These are typically diseases that already have an indication for biopsy, such as leprosy, vasculitis, amyloidosis and acquired demyelinating diseases. Nerve biopsies helped to clarify differential diagnoses, including neuritis versus leprosy, amyloidosis versus leprosy, vasculitis versus leprosy and vasculitis versus neuritis. They were also essential for the diagnosis of PNL in cases of multiple mononeuropathy, as the patient does not present clear clinical signs of leprosy (especially no pain, no change in sensory, no thickening of the nerve and no skin lesion).

## Conclusion

Nerve biopsy remains a valuable tool for reaching a definitive diagnosis, ruling out differential diagnoses of some peripheral neuropathies, and guiding treatment in uncertain cases. We recommend considering the exam in cases after an extensive clinical investigation not confirm the diagnosis to optimize its usefulness, especially in cases of asymmetric clinical presentation and progressive deterioration.

Future diagnostic strategies will likely benefit from the integration of nerve biopsy with high-sensitivity molecular tools and advanced imaging modalities, allowing tissue-level insights to complement non-invasive approaches and improve diagnostic precision in challenging neuropathies. The application of molecular techniques, including PCR and sequencing-based assays, may enhance pathogen detection and improve diagnostic sensitivity when applied to biopsy specimens. Rather than being replaced by emerging technologies, nerve biopsy is likely to remain a complementary method that provides essential morphological and immunopathological information. The combined use of these modalities may contribute to more accurate disease characterization, improved diagnostic efficiency and better-informed therapeutic decision-making.

## Supplementary Material

fcag249_Supplementary_Data

## Data Availability

The datasets collected and/or analysed during the present study are available from the corresponding author upon reasonable request.
