# Supplementary material for: Peripheral nerve biopsy in pure neural leprosy: a 26-year experience in Brazil
Source: Brain Commun. 2026 Jun 26;8(4):fcag249. doi: 10.1093/braincomms/fcag249 (PMC13348849; doi:10.1093/braincomms/fcag249)
Supplement: fcag249_Supplementary_Data [file fcag249_supplementary_data.zip › Supplementary Table 1.docx]

**Supplementary Table 1. Histopathological parameters evaluated in nerve biopsy**

| HISTOPATHOLOGICAL PARAMETERS | |
| --- | --- |
| PARAFFIN SECTION | **SEMITHIN SECTION** |
| Architecture change | Decrease in the number of large myelin fibers |
| Number of fascicles in paraffin sections | Decrease in the number of small myelin fibers |
| Number of fascicles in semithin sections | Demyelination |
| Number of fascicles with inflammatory infiltrate | Axonal degeneration |
| Percentage of fascicles with inflammatory infiltrate | Remyelination |
| Degree of inflammatory infiltrate in epineurium | Axonal regeneration |
| Degree of inflammatory infiltrate in the perineurium |  |
| Degree of endoneurial inflammatory infiltrate |  |
| Degree of lymphohistiocytic inflammatory infiltrate |  |
| Epithelioid granuloma |  |
| Vacuolated macrophages |  |
| Caseous necrosis |  |
| Degree of epineural fibrosis |  |
| Degree of perineural fibrosis |  |
| Degree of endoneurial fibrosis |  |
| Decrease in the number of neural fibers |  |
| Endoneurial edema |  |
| Schwann cell proliferation |  |
| Pericyte hyperplasia |  |
| AFB: degree of bacilli; intact or granular bacilli |  |
| Myelin ovoid |  |
| Angiogenesis |  |
| Vasculitis |  |
| Hyaline-vascular thickening |  |
| Perineural hyperplasia |  |
