# Supplementary figures and images for: Peripheral nerve biopsy in pure neural leprosy: a 26-year experience in Brazil
Source: Brain Commun. 2026 Jun 26;8(4):fcag249. doi: 10.1093/braincomms/fcag249 (PMC13348849; doi:10.1093/braincomms/fcag249)

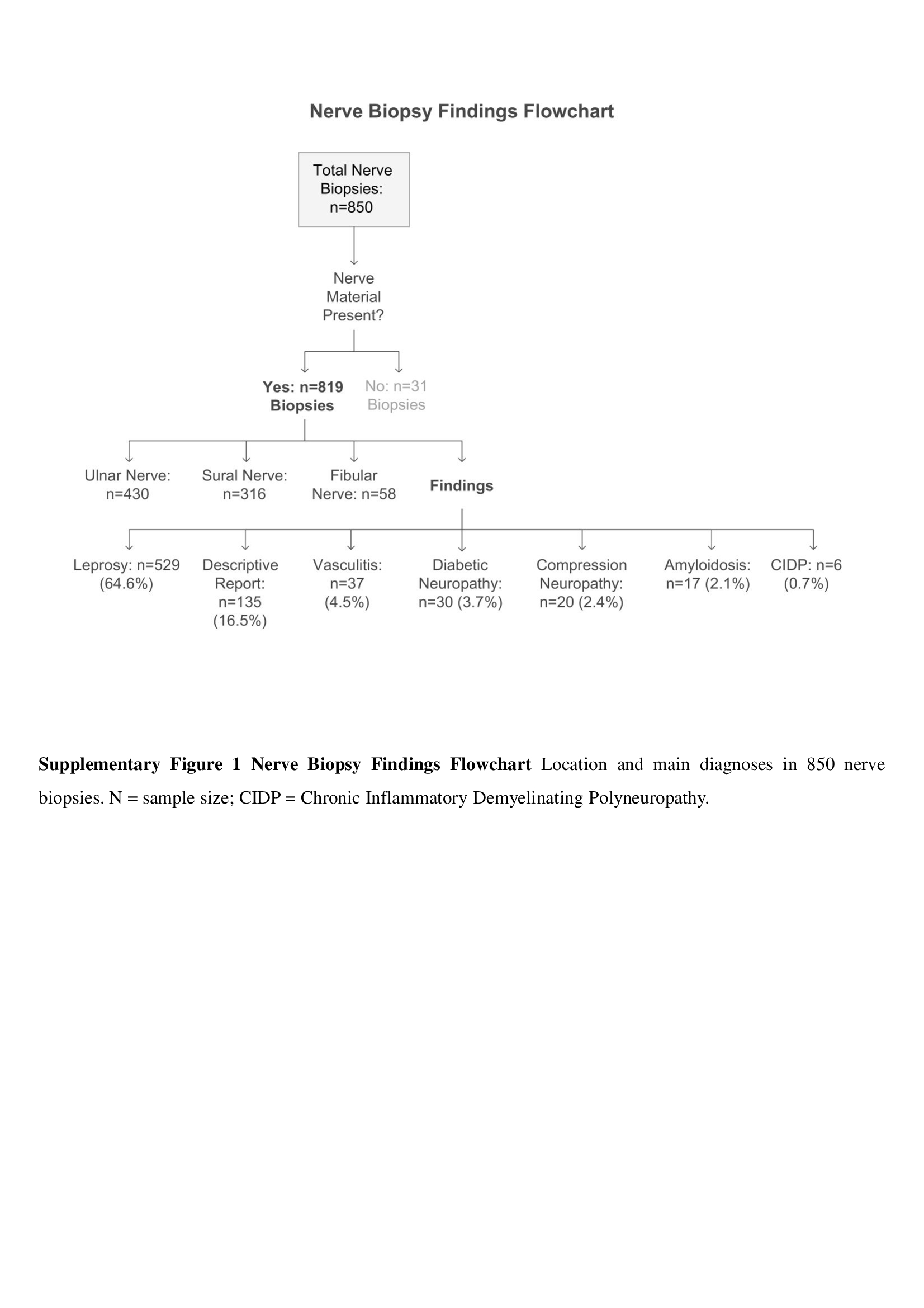

Supplement: fcag249_Supplementary_Data [file fcag249_supplementary_data.zip › Supplementary_Figure_1.tif]
